# QRS 3D voltage-time integral in narrow QRS complex – Establishing the normal reference range

**DOI:** 10.1016/j.jelectrocard.2025.154069

**Published:** 2025-07-21

**Authors:** Amulya Gupta, Christopher J. Harvey, Uzair Mahmood, Jacob Baer, Nikhil Parimi, Ashutosh Bapat, Seth H. Sheldon, Madhu Reddy, Zijun Yao, Yongkuk Lee, Amit Noheria

**Affiliations:** a Department of Cardiovascular Medicine, University of Kansas Medical Center, Kansas City, KS, United States; b Department of Cardiovascular Medicine, Westchester Medical Center, Valhalla, NY, United States; c Department of Cardiovascular Medicine, University Hospitals Cleveland Medical Center, Cleveland, OH, United States; d Department of Electrical Engineering and Computer Science, University of Kansas, Lawrence, KS, United States; e Department of Biomedical Engineering, Wichita State University, Wichita, KS, United States

**Keywords:** Vectorcardiography, Voltage time integral, QRS area, Electrocardiogram, VTI, Cardiomyopathy, 3D QRS, Reference range

## Abstract

**Background:**

Vectorcardiographic 3D QRS voltage-time integral (VTI_QRS-3D_) is a novel marker of ventricular dyssynchrony pertinent for cardiac resynchronization therapy. It may have additional clinical utility but its normal reference ranges have not been established. We sought to define reference ranges for VTI_QRS-3D_ in healthy individuals.

**Methods:**

We retrospectively analyzed 12‑lead ECGs of healthy adults (2010–2014) and compared them to patients with cardiomyopathy with reduced ejection fraction (EF) <50 %. Using the Kors matrix, 12‑lead ECGs with QRS duration ≤120 ms were converted to vectorcardiographic X, Y, and Z leads. VTI_QRS-3D_ was calculated as the instantaneous root-sum-square (3D) voltage integrated over the QRS duration. Reference range limits were defined as the 2.5th to 97.5th percentiles respectively for healthy females and males in age groups 18–34, 35–54 and ≥ 55 years.

**Results:**

The study included 468 healthy adults (age 44.6 ± 17.0 years; 63.9 % female) and 314 patients with cardiomyopathy (age 62.1 ± 14.0 years; 34.4 % female). VTI_QRS-3D_ was significantly larger in the cardiomyopathy patients compared to the healthy population (48.2 ± 21.4 vs. 38.1 ± 9.3 μVs, *p* < 0.0001). Increased age and female sex were significant predictors of lower VTI_QRS-3D_ in the healthy population (both p < 0.0001) VTI_QRS-3D_ reference ranges for respective age groups for healthy females were 23.2–55.0, 23.9–56.4 and 19.6–50.9 μVs, and for healthy males were 29.9–57.2, 28.2–56.7 and 21.4–55.9 μVs.

**Conclusion:**

VTI_QRS-3D_ is higher in younger individuals and males within healthy adult population but is overall higher in patients with cardiomyopathy with reduced EF. Age and sex need to be accounted for using VTI_QRS-3D_ as a marker for cardiac disease.

## Introduction

**Fig. 1.**
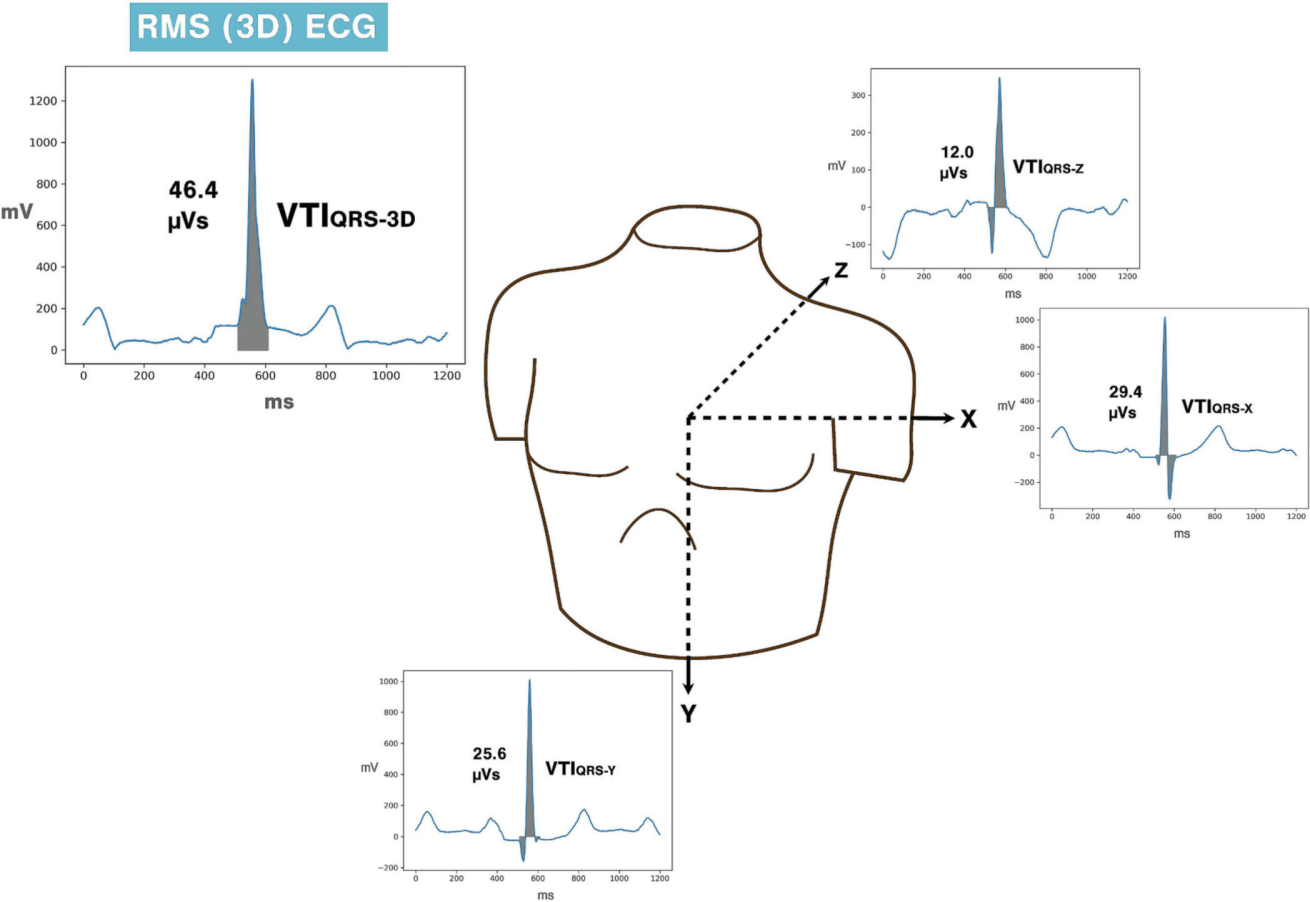
Schematic representation of VTI_QRS-3D_ calculation.

Electrocardiograms (ECGs) are recorded using a standard 12‑lead configuration, which includes 3 limb and 6 precordial electrodes. While this 12‑lead ECG format is widely used and supported by over a century of research, it represents cardiac electrical activity along anatomically arbitrary axes rather than providing a true three-dimensional (3D) depiction of cardiac electrical activity. Vectorcardiography (VCG) addresses this limitation by representing cardiac electrical activity along the orthogonal cartesian axes X (right-to-left), Y (cranial-to-caudal), and Z (anterior-to-posterior) [1]. Although VCG is seldom performed in contemporary clinical practice, it can be derived from a 12‑lead ECG using various transformation matrices such as Kors’s or Dower’s regression matrices [2]. Plotting the root-sum-square (RMS) of the instantaneous voltages of X, Y, and Z leads yields the 3D ECG, which is a scalar representation of the net surface cardiac voltages (Fig. 1).

Recent work has established voltage-time integral of the 3D ECG QRS complex (VTI_QRS-3D_) and a related metric, 3D-QRS area, as novel markers of left ventricular electrical dyssynchrony [3,4]. By integrating the instantaneous 3D voltage over the duration of QRS complex, VTI_QRS-3D_ quantifies the total ECG potentials recorded during ventricular depolarization. Mechanistically, electrical dyssynchrony fragments the depolarization wavefront, disrupting the cancellation of opposing synchronized wavelets and leads to increased electrical potentials which manifests as higher VTI_QRS-3D_ [5,6]. VTI_QRS-3D_ has proven to be a stronger predictor of response to cardiac resynchronization therapy (CRT) compared to QRS duration [4]. Its utility extends beyond CRT response, as previous studies have also demonstrated the application of VTI_QRS-3D_ in identifying left ventricular hypertrophy [7,8].

Despite its advantages, VTI_QRS-3D_ is not routinely reported in clinical ECGs, and limited literature exists regarding its normal range beyond the contexts of CRT and left ventricular hypertrophy. This study aims to establish a reference range for VTI_QRS-3D_ in healthy patients without cardiac electrical or structural disease. Specifically, we evaluated VTI_QRS-3D_ in healthy individuals with normal ECGs and narrow QRS complexes, and compared to patients having cardiomyopathy with reduced ejection fraction (EF) and normal QRS duration.

## Methods

This study was conducted under approval by the Institutional Review Board at The University of Kansas. We conducted a retrospective analysis on ECGs from 2010 to 2014 at The University of Kansas Medical Center (KUMC). This retrospective cohort consisted of two populations with narrow QRS complexes (≤120 ms). The first population, referred to as ‘healthy group’, consisted of individuals lacking a history of cardiomyopathy and ECG conduction abnormalities while the second population, referred to as ‘cardiomyopathy group’, consisted of patients with a known diagnosis of cardiomyopathy with reduced EF and a narrow QRS complex.

The healthy population was queried using Healthcare Enterprise Repository for Ontological Narration (HERON), which is a repository of all health visit International Classification of Diseases (ICD) codes combined with a variety of hospital and medical center electronic records [9,10]. We identified patients with an outpatient routine preventative health visit code and a procedure code for ECG between 2010 and 2014. We excluded patients with diagnostic codes for any cardiovascular disease or chronic non-communicable disease diagnosis. We then manually downloaded their digital ECG files in .xml and .pdf formats from the Philips^®^ IntelliSpace^™^ ECG management system. All ECGs with QRS duration >120 ms were excluded. The ECGs were then reviewed by an experienced electrophysiologist (AN) for any abnormal findings. Patient charts were then manually reviewed to identify any cardiovascular or physical disease condition, upon identification of which these ECGs were also excluded.

The cardiomyopathy with reduced EF patients were also queried using HERON to identify echocardiographic left ventricular EF below 50 %. The cardiomyopathy group was further classified into ischemic and nonischemic cardiomyopathy based on ICD 10 codes for cardiomyopathy (I42) and chronic ischemic heart disease (I25). Structured clinical echocardiographic reports were used to extract left ventricular dimensions and EF which were measured according to the American Society of Echocardiography guidelines [11]. At our center, the EF is calculated by Biplane Simpson’s method using echo contrast if needed. In this group, patients with a history of cardiac arrhythmias or conduction abnormalities were excluded.

### ECG processing:

Clinical 12‑lead ECG .xml files were retrieved from the Philips^®^ IntelliSpace^™^ ECG management system and processed using Python. The 12‑lead 1200 ms representative beat ECG signals were converted to orthogonal X, Y, Z leads using the Kors conversion matrix [12]. RMS of the orthogonal leads was computed to generate a 3D ECG signal. The location of QRS onset and QRS duration were obtained from the proprietary Philips DXL algorithm. VTI_QRS-3D_ was obtained by integrating the voltage across the QRS complex. Similarly, individual VTI_QRS_ for X, Y, Z leads were also calculated (Fig. 1).

### Statistical analysis:

Continuous variables were expressed as mean ± standard deviation (SD) and categorical variables as n (%). VTI_QRS-3D_ values were reported using mean ± SD, median and percentiles (2.5th, 25th, 75th and 97.5th). Reference ranges were defined as values between 2.5th and 97.5th percentiles. Continuous variables were compared using independent sample *t*-test and one way ANOVA, and categorical variables were compared using the χ2 test. Univariate and multivariate linear regression was used to assess association between predictor variables and VTI_QRS-3D_, with results expressed as a β-coefficient ± standard error (SE). All statistical analyses were done in JMP Pro 17 (SAS Inst. Cary, NC, USA) and R (R version 4.4.1).

## Results

The healthy group included 468 adults. The QRS duration in this healthy population was 86.9 ± 9.7 ms. The VTI_QRS_ in the vectorcardiographic X, Y, Z leads was 23.8 ± 7.1 μVs, 19.1 ± 7.7 μVs and 15.4 ± 7.1 μVs respectively. The VTI_QRS-3D_ among this healthy group was 38.1 ± 9.3 μVs. The cardiomyopathy with reduced EF group included 314 patients. The baseline demographic, echocardiographic and ECG variables for both groups are summarized in Table 1.

### Baseline demographics:

In the healthy group, 299 (63.9 %) were female, with a mean age of 44.6 ± 17.0 years, and 312 (66.7 %) identified as white. In the cardiomyopathy group, 108 (34.4 %) were female, the mean age was 62.1 ± 14.0 years, and 197 (62.7 %) identified as white.

All the baseline demographic variables differed significantly between the groups (all *p* < 0.05).

### Echocardiogram variables:

Echocardiographic variables were available for 132 (27.8 %) patients in the healthy group, and all patients in the cardiomyopathy group. The healthy group demonstrated smaller left ventricular internal dimensions in diastole (LVIDd: 4.4 ± 0.6 cm vs. 5.3 ± 0.8 cm) and systole (LVIDs: 2.9 ± 0.5 cm vs. 4.2 ± 0.9 cm) compared to the cardiomyopathy group (both *p* < 0.0001). EF was higher in the healthy group (59.5 ± 3.5 vs. 35.9 ± 9.4, *p* < 0.0001). Additionally, the healthy group had lower left ventricular mass indexed (LVMi: 68.8 ± 16.6 g/m^2^ vs. 110.6 ± 36.1 g/m^2^, *p* < 0.0001), interventricular septum thickness (0.9 ± 0.2 cm vs. 1.1 ± 0.2 cm, p < 0.0001), and left ventricular posterior wall thickness (0.9 ± 0.2 cm vs. 1.1 ± 0.2 cm, *p* < 0.0001).

### ECG variables:

The healthy group had shorter QRS duration compared to the cardiomyopathy group (86.9 ± 9.7 ms vs. 94.1 ± 11.4 ms, *p* < 0.0001). Among the VCG variables, the healthy group had smaller Amplitude_QRS-3D_ (1.27 ± 0.37 mV vs. 1.34 ± 0.62 mV, *p* = 0.03), VTI_QRS-X_ (23.8 ± 7.1 μVs vs. 28.0 ± 16.0 μVs, p < 0.0001), VTI_QRS-Z_ (15.4 ± 7.1 μVs vs. 25.7 ± 14.6 μVs, p < 0.0001), and VTI_QRS-3D_ (38.1 ± 9.3 μVs vs. 48.2 ± 21.4 μVs, *p* < 0.0001). VTI_QRS-Y_ was similar between both groups (19.1 ± 7 0.7 μVs vs. 19.5 ± 12.2 μVs, *p* = 0.6).

### Vectorcardiographic lead VTI_QRS_ in healthy group:

The distribution of VTI_QRS_ for vectorcardiographic X, Y and Z leads for the healthy group are shown in Table 2A–2B. The VTI_QRS_ values in X and Z leads are smaller for females compared to males (both *p* <0.0001), while VTI_QRS-Y_ shows no sex-based difference (*p* = 0.8). In general, the VTI_QRS_ for Y (*p* < 0.0001) and X (*p* = 0.02) leads decreases with increasing age, while VTI_QRS-Z_ does not show any age-related trend.

### 3D RMS ECG VTI_QRS_ in healthy group:

**Fig. 2.**
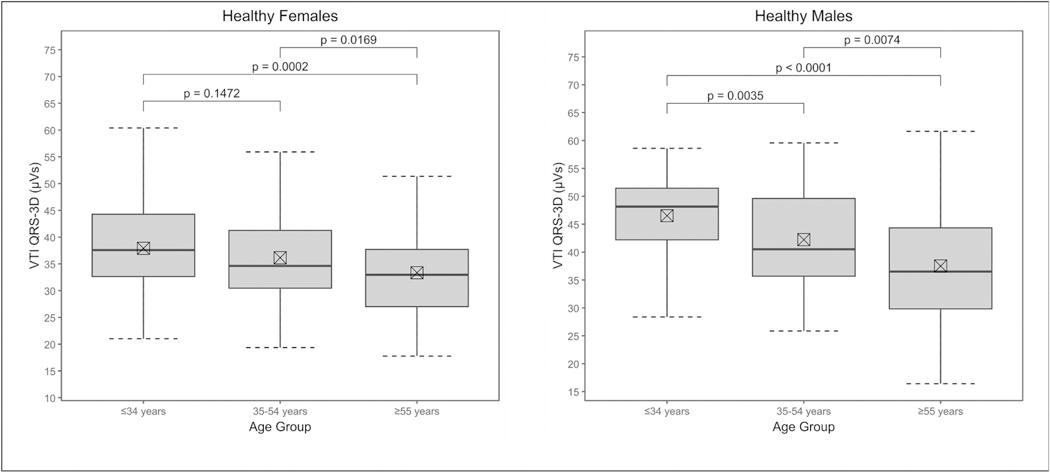
Boxplots for VTI_QRS-3D_ by age groups for healthy females (n = 299) and healthy males (n = 169).

The distribution of VTI_QRS-3D_ among different demographic categories are summarized in Table 3A for females and Table 3B for females. Briefly, VTI_QRS-3D_ showed a decreasing trend across age groups from 18 to 65 years (*p* = 0.003 for females, p < 0.0001 for males). The values of VTI_QRS-3D_ were similar across racial groups (*p* = 0.2 females, *p* = 0.5 males), and did not exhibit statistically significant association with body surface area (BSA, p = 0.5 females, *p* = 0.08 males) or body mass index (BMI, p = 0.2 females, p = 0.08 males). The distribution of VTI_QRS-3D_ across age groups for healthy population is summarized in Fig. 2.

Echocardiographic variables were available for 88 (29.4 %) females and 44 (26.0 %) males. These patients had normal echocardiographic values with small variability. In this relatively homogenous population, the echocardiographic variables exhibited no statistical associations with VTI_QRS-3D_ for females and only had minor statistical significance in males for LVIDd (univariate β =5.0 ± 2.2, *p* = 0.03) and EF (univariate β = − 0.9 ± 0.4, *p* = 0.01).

As shown in Table 4, in the healthy group, the statistically significant multivariate predictors of VTI_QRS-3D_ were age (β = − 0.14 ± 0.02, *p* < 0.0001) and female sex (β = − 6.41 ± 0.91, p < 0.0001).

### Cardiomyopathy group:

Variables for females and males in the cardiomyopathy group are summarized in Tables 5A and 5B. In females, VTI_QRS-3D_ was comparable across age groups, with no significant differences noted (*p* = 0.9). Similarly, values were comparable across racial groups (*p* = 0.3) and cardiomyopathy types (ischemic vs. non-ischemic, p = 0.3). In males, VTI_QRS-3D_ showed no significant age-related trends (*p* = 0.2), but there were differences across racial groups (*p* = 0.007) and between ischemic and non-ischemic cardiomyopathy types (44.3 ± 17.3 μVs vs. 54.4 ± 21.2 μVs, *p* = 0.0003). Among echocardiographic variables, VTI_QRS-3D_ was positively associated with LV dimensions and calculated LVMi. VTI_QRS-3D_ was negatively associated with EF in females (β = − 0.7 ± 0.3, *p* = 0.01) with a similar but weaker trend in males (β = − 0.3 ± 0.1, *p* = 0.08). In the cardiomyopathy group, non-ischemic cardiomyopathy (β =6.74 ± 2.46, *p* = 0.006) and LVMi (β =0.25 ± 0.03, *p* < 0.0001) were significant multivariate predictors of VTI_QRS-3D_ (Table 6).

### Reference ranges:

The overall reference range (2.5th–97.5th percentiles) for the entire healthy population is 20.9–56.4 μVs. The reference range of VTI_QRS-3D_ specifically for females is 20.2–55.7 μVs and for males is 25.6–57.2 μVs. The percentile values of VTI_QRS-3D_ among healthy group for various age groups and sex are shown in Table 7. The reference ranges by age groups for females were 23.2–55.0 μVs (18–34 years), 23.9–56.4 μVs (35–54 years), and 19.6–50.9 μVs (≥55 years). For males, the ranges were 29.9–57.2 μVs, 28.2–56.7 μVs, and 21.4–55.9 μVs, respectively

## Discussion

In this study, we computed the reference values for VTI_QRS-3D,_ an automatically calculable measurement with potential for integration into automated ECG analysis. Several features of VTI_QRS-3D,_ including robust automated calculation and efficient summarization of myocardial depolarization in one numerical value, make it a suitable metric for ECG-based research and broader assessment of clinical applications. As opposed to the QRS duration, which has high interobserver and interobserver variability in measurement, the VTI_QRS-3D_ is very reproducible as it weights the QRS duration to the 3D/RMS voltage at any instant during the QRS, thereby assigning very small weights to the beginning and end of QRS. On the other hand, as opposed to QRS voltage alone, VTI does incorporate the QRS duration and is therefore a more accurate representation of the summed force of the ventricular activation ECG potential. The average VTI_QRS-3D_ in our healthy group was 38.1 ± 9.3 μVs and among cardiomyopathy with reduced EF patients was 48.2 ± 21.4 μVs.

### Terminology disambiguation:

The literature contains various terms to describe VTI_QRS-3D_ and related measurements. One commonly used metric is 3D QRS area (or QRS_AREA_), which is derived by calculating the root-sum-square of the individual voltage-time integrals of QRS projections along the X, Y and Z axes, and has been used in studies evaluating CRT response [3,13]. 3D QRS area differs from VTI_QRS-3D_, where voltage-time integral is calculated from the scalar 3D lead obtained by plotting root-sum-square of the X, Y and Z leads. Further, 3D QRS area can be calculated using two methods: the summation method and the difference method. In the summation method, areas under the positive and negative deflections along each lead are added, while in the difference method, they are subtracted. We have shown previously that the values of 3D QRS area obtained through the summation method are close to the VTI_QRS-3D_ values in normal ECGs (linear regression, β 1.07, R^2^ 0.99), while those obtained using the difference method can diverge significantly (β 1.42, R^2^ 0.65) [14]. In older studies, spatial vector of QRS (SÂ QRS) has been used by Pipberger et al. to describe 3D QRS area obtained via the difference method [15,16]. Of note, they also included P-wave integrals in this metric, assuming the P-wave contribution to be negligible. Tereshchenko et al. introduced the sum absolute QRST integral (SAI QRST), which is calculated using the arithmetic sum of orthogonal lead areas (derived via the summation method) instead of the root-sum-square used for the 3D QRS area and includes the T-wave in its computation [17]. Later, the term SAI QRS has been used as well, which does not incorporate the T-wave [18]. Although these interrelated metrics differ in their calculation, their general associations with clinical covariates are expected to remain similar.

### Trends in VTI_QRS-3D_:

We noted several trends of VTI_QRS-3D_ with covariates. Foremost, in the healthy group, older age and female sex were associated with smaller values of VTI_QRS-3D_. Notably, age stratification revealed that the negative correlation between age and VTI_QRS-3D_ persists up to approximately 65 years of age, beyond which it stabilizes or may even show a slight increase. A decrease in SAI QRS and SÂ QRS with age in healthy subjects has been observed in previous studies as well [15,18]. The mechanism for this remains unclear although this may be attributable to cardiac atrophy that occurs with age [19]. Beyond 65 years, increased incidence of asymptomatic structural heart disease and conduction abnormalities may explain the stabilization or increase noted in VTI_QRS-3D_ values. Our finding of smaller VTI_QRS-3D_ in females is consistent with previous studies [7]. The average cardiac mass is lower in females, which likely contributes to a reduced QRS voltage and reduced QRS duration, and thus a lower VTI_QRS-3D_ [20,21]. Females have a smaller BSA that may correlate with a smaller cardiac mass to reduce VTI_QRS-3D_, but also less dampening of the cardiac voltage from a smaller thorax that may increase VTI_QRS-3D_. Though we didn’t find any association between BSA or BMI in within the healthy female or male subgroups, a previous study reported a positive sex-adjusted correlation between BSA and VTI_QRS-3D_ [22]. Further, breast tissue may attenuate precordial QRS amplitudes, though evidence suggests this effect is negligible [23]. Nevertheless, these observations underscore the need to use different sex-specific VTI_QRS-3D_ cut-offs.

Second, patients having cardiomyopathy with reduced EF had significantly larger VTI_QRS-3D_ as compared to healthy patients. This is an expected finding, since cardiomyopathy with reduced EF is associated with ventricular activation delay and increased LV mass/volume, which may lead to prolonged QRS duration and increased QRS voltage [24–26]. Previous studies show that cardiac resynchronization therapy (CRT) response is better in patients with larger 3D QRS area, since it reflects delayed LV activation which can be mitigated by CRT [3]. Furthermore, VTI_QRS-3D_ increases with LV mass and serves as a superior ECG predictor of left ventricular hypertrophy, when compared to previously published voltage-based criteria [7,27].

Third, in the cardiomyopathy with reduced EF group, patients with non-ischemic as opposed to ischemic cardiomyopathy and higher left ventricular mass indexed (LVMi) had larger VTI_QRS-3D_. The association between non-ischemic cardiomyopathy and increased 3D QRS area has also been observed in multiple previous studies and is probably related to a higher LVMi due to LV dilation in non-ischemic patients and loss of QRS voltage due to infarcted myocardium among ischemics [13,28,29]. Interestingly, unlike in the healthy group, age and sex did not show statistically significant associations with VTI_QRS-3D_ in patients having cardiomyopathy with reduced EF. As the cardiomyopathy group was predominantly composed of older patients (70 % aged ≥55 years), the relatively constrained age distribution may have limited the ability to detect a statistically significant association between age and VTI_QRS-3D_. On the other hand, this could suggest that specific characteristics of cardiomyopathy, such as underlying mechanisms and increased LV mass, may serve as dominant drivers or effect modifiers, obfuscating the effect of age and sex on VTI_QRS-3D_ in this population.

### Previously published reference ranges:

Pipberger et al. published age-based reference ranges of SÂ QRS for 518 normal men in 1967 [15]. Similar to our results, they observed a decline in SÂ QRS with age, with mean values ranging from 42.0 ± 13.0 μVs in 20–29 years group to 32.4 ± 13.4 μVs in the 60–78 years group. In our sample, the mean VTI_QRS-3D_ values in healthy men were shifted higher, from 46.5 ± 7.6 μVs in 18–34 years group to 38.4 ± 10.4 μVs in ≥65 years group. The differences in Pipberger et al. and our magnitudes may be accounted by the differences in the calculation of SÂ QRS versus VTI_QRS-3D_. The SÂ QRS metric of Pipberger et al. is similar to 3D QRS area calculated using the difference method, which is a systematic underestimate of VTI_QRS-3D_ [14]. Further, Pipberger et al. used the original Frank orthogonal lead system to record vectorcardiograms, whereas we derived orthogonal leads from standard 12‑lead ECGs using the Kors matrix [1]. We have provided a comparison of the data by Pipberger et al. vis-à-vis 3D QRS area using the difference method from our dataset in Supplementary Table 1.

More recently, in a comprehensive analysis of various vectorcardiography parameters, De la Garza Salazar and Egenriether studied mean values of SAI QRS for different categories of age, sex, BMI, hypertension, ischemic heart disease, and left ventricular hypertrophy [18]. Owing to differences in calculation of these metrics (we took the time integral of instantaneous RMS voltage while they took the arithmetic sum of the integrals in X, Y and Z leads), we observed overall mean VTI_QRS-3D_ value of approximately 40 μVs, whereas they observed mean SAI QRS values closer to 60 μVs across groups (Supplementary Table 2). Similar to our results, they observed a decrease in SAI QRS values with increased age and female sex.

### Strengths and limitations:

The main strength of our analysis is the delineation of a healthy population verified through a manual chart review, ensuring that ECGs in the healthy group belong to patients without pre-existing cardiovascular disease. Our analysis encompassed a diverse population including various ages, sexes and races. However, there are several important limitations to our analysis. These include a limited sample size, limited racial and ethnic diversity, and the use of clinical ECGs with potential to introduce bias rather than seeking ECGs from healthy volunteers in the community.

## Conclusions

VTI_QRS-3D_ is higher in younger individuals and males within a healthy adult population but is overall higher in patients with cardiomyopathy with reduced EF. If adopted for future clinical reporting, VTI_QRS-3D_ reference ranges should be interpreted in the context of these predictors. Further, there is a need to standardize terminology and computation algorithms for VTI_QRS-3D_ and related QRS area metrics.

## Supplementary Material

1

Appendix A. Supplementary data

Supplementary data to this article can be found online at https://doi.org/10.1016/j.jelectrocard.2025.154069.

## Figures and Tables

**Table 1:** Baseline characteristics in the normal and cardiomyopathy with reduced EF populations.

Variables	Healthy group *(n* = *468)*	Cardiomyopathy group *(n* = *314)*	p-value

**Demographics**			
Age, years	44.6 ± 17.0	62.1 ± 14.0	**<0.0001**
Women	299 (63.9 %)	108 (34.4 %)	**<0.0001**
Race			
White	312 (66.7 %)	197 (62.7 %)	
Black	73 (15.6 %)	79 (25.2 %)	**0.001**
Other	83 (17.7%)	38 (12.1 %)	
Body surface area, m^2^	1.85 ± 0.27	1.98 ± 0.31	**<0.0001**
**Echocardiogram**	*n* = *132*		
LV internal dimension in diastole, cm	4.4 ± 0.6	5.3 ± 0.8	**<0.0001**
LV internal dimension in systole, cm	2.9 ± 0.5	4.2 ± 0.9	**<0.0001**
LV ejection fraction, %	59.5 ± 3.5	35.9 ± 9.4	**<0.0001**
LV mass, g	127.1 ± 38.6	219.6 ± 79.9	**<0.0001**
LV mass indexed, g/m^2^	68.8 ± 16.6	110.6 ± 36.1	**<0.0001**
Interventricular septum, cm	0.9 ± 0.2	1.1 ± 0.2	**<0.0001**
LV posterior wall, cm	0.9 ± 0.2	1.1 ± 0.2	**<0.0001**
**ECG**			
QRS duration, ms	86.9 ± 9.7	94.1 ± 11.4	**<0.0001**
Amplitude_QRS-3D,_ mV	1.27 ± 0.37	1.34 ± 0.62	**0.03**
VTI_QRS-3D,_ μVs	38.1 ± 9.3	48.2 ± 21.4	**<0.0001**
VTI_QRS-X,_ μVs	23.8 ± 7.1	28.0 ± 16.0	**<0.0001**
VTI_QRS-Y,_ μVs	19.1 ± 7 0.7	19.5 ± 12.2	0.6
VTI_QRS-Z,_ μVs	15.4 ± 7.1	25.7 ± 14.6	**<0.0001**

**Table 2A:** Distributions of VTI_QRS_ in vectorcardiographic X, Y, Z leads in healthy females (*n* = 299).

Variable	Mean ± S.D. (Healthy females)		
VTI_QRS,_ μVs	X	Y	Z

**Overall**	22.0 ± 6.1	19.0 ± 7.6	14.0 ± 6.4
**Age, years**	**p = 0.02**	**p < 0.0001**	p = 0.9
18–34	22.8 ± 5.7	21.7 ± 7.9	14.0 ± 6.6
35–54	22.7 ± 6.8	18.8 ± 7.2	13.9 ± 6.9
55–64	20.6 ± 4.9	16.9 ± 6.8	13.6 ± 5.6
≥65	20.2 ± 6.2	16.4 ± 7.4	14.6 ± 6.1
**Race**	p = 0.3	***p* = 0.04**	*p* = 0.3
White	21.9 ± 6.3	18.6 ± 7.4	13.9 ± 6.3
Black	23.1 ± 5.9	21.3 ± 8.6	13.3 ± 6.0
Other/unknown	21.2 ± 5.3	17.9 ± 6.7	15.2 ± 7.3
**Body Composition**	**β ± SE (*p*-value)**		
Body surface area, m^2^	2.0 ± 1.6 *(p* = 0.2)	−1.4 ± 2.0 *(p* = 0.5)	2.4 ± 1.6 *(p* = 0.1)
Body mass index, kg/m^2^	−0.05 ± 0.06 (*p* = 0.4)	−0.18 ± 0.08 **(*p* = 0.02)**	0.06 ± 0.06 (*p* = 0.3)

**Table 2B:** Distributions of VTI_QRS_ in vectorcardiographic X, Y, Z leads in healthy males (*n* = 169).

Variable	Mean ± S.D. (Healthy males)		
VTI_QRS,_ μVs	X	Y	Z

**Overall**	26.9 ± 7.5	19.2 ± 8.0	18.0 ± 7.5
**Age, years**	**p = 0.02**	**p < 0.0001**	*p* = 0.09
18–34	29.0 ± 6.9	23.0 ± 7.3	18.6 ± 7.9
35–54	26.7 ± 7.1	18.2 ± 7.6	19.2 ± 7.8
55–64	23.8 ± 8.3	16.4 ± 7.7	15.3 ± 5.7
≥65	26.2 ± 7.6	15.5 ± 7.4	16.6 ± 7.1
**Race**	p = 0.5	*p* = 0.8	p = 0.3
White	26.8 ± 7.2	19.0 ± 8.2	17.5 ± 7.7
Black	28.8 ± 7.5	20.4 ± 5.3	17.7 ± 7.1
Other/unknown	26.3 ± 8.3	19.2 ± 8.6	19.6 ± 7.3
**Body Composition**	**β ± SE (*p*-value)**		
Body surface area, m^2^	1.0 ± 2.0 *(p* = 0.6)	−4.9 ± 2.1 (**p = 0.02**)	3.5 ± 2.0 *(p* = 0.09)
Body mass index, kg/m^2^	−0.05 ± 0.11 (p = 0.6)	−0.32 ± 0.12 (***p* = 0.007**)	−0.16 ± 0.11 (p = 0.2)

**Table 3A:** Distribution of VTI_QRS-3D_ among healthy females.

Variables (Healthy females)	n	VTI_QRS-3D,_ μVs (Mean ± SD)	IQR	Reference ranges 2.5th-97.5th percentile	p-value

**Overall**	299	35.7 ± 8.6	29.6–41.4	20.2–55.7	–
**Age, years**					**0.003**
18–34	92	37.9 ± 8.5	32.6–44.3	23.2–55.0	
35–54	107	36.1 ± 8.6	30.5–41.2	23.9–56.4	
55–64	58	33.3 ± 7.5	28.2–36.9	22.3–51.0	
≥65	42	33.4 ± 8.9	25.5–39.3	19.1–46.8	
**Race**					0.2
White	201	35.3 ± 8.6	29.6–40.7	20.1–54.6	
Black	53	37.6 ± 8.9	30.8–43.1	24.5–55.7	
Other/unknown	45	35.4 ± 8.2	30.1–41.6	21.2–49.6	
**Body Composition**		**Mean ± SD**	**β-coefficient**		
Body surface area, m^2^	298	1.8 ± 0.2	−1.5 ± 2.2		0.5
Body mass index, kg/m^2^	298	26.1 ± 5.8	−0.1 ± 0.1		0.2
**Echocardiography**		**Mean ± SD**	**β-coefficient**		
LV internal dimension in diastole, cm	88	4.3 ± 0.6	2.4 ± 1.7		0.2
LV internal dimension in systole, cm	88	2.8 ± 0.4	3.8 ± 2.2		0.08
LV ejection fraction, %	88	59.8 ± 3.5	−0.03 ± 0.3		0.9
LV mass, g	88	113.4 ± 27.8	0.02 ± 0.03		0.5
Interventricular septum, cm	88	0.83 ± 0.14	−10.0 ± 6.9		0.1
LV posterior wall, cm	88	0.84 ± 0.12	−5.0 ± 7.9		0.5

**Table 3B:** Distribution of VTI_QRS-3D_ among healthy males.

Variables (Healthy males)	n	VTI_QRS-3D,_ μVs Mean ± SD	IQR	Reference ranges 2.5th–97.5th percentile	p-value

**Overall**	169	42.3 ± 9.2	35.4–49.9	25.6–57.2	**–**
**Age, years**					**<0.0001**
18–34	58	46.5 ± 7.6	42.2–51.5	29.9–57.2	
35–54	61	42.2 ± 8.1	35.7–49.6	28.2–56.7	
55–64	31	37.0 ± 9.4	29.2–41.9	25.1–56.7	
≥65	19	38.4 ± 10.4	32.9–45.5	18.3–53.9	
**Race**					0.5
White	111	41.8 ± 9.0	35.3–49.3	26.4–57.3	
Black	20	44.4 ± 9.5	39.2–51.2	24.0–55.5	
Other/unknown	38	42.8 ± 9.5	35.3–50.2	25.6–57.0	
**Body Composition**		**Mean ± SD**	**β-coefficient**		
Body surface area, m^2^	167	2.0 ± 0.3	−4.3 ± 2.4		0.08
Body mass index, kg/m^2^	167	26.5 ± 5.2	−0.2 ± 0.1		0.08
**Echocardiography**		**Mean ± SD**	**β-coefficient**		
LV internal dimension in diastole, cm	44	4.7 ± 0.6	5.0 ± 2.2		**0.03**
LV internal dimension in systole, cm	44	3.0 ± 0.5	4.6 ± 2.8		0.1
LV ejection fraction, %	44	59.1 ± 3.5	−0.9 ± 0.4		**0.01**
LV mass, g	44	154.5 ± 42.7	0.04 ± 0.03		0.2
Interventricular septum, cm	44	0.96 ± 0.20	−4.1 ± 6.5		0.5
LV posterior wall, cm	44	0.95 ± 0.16	3.6 ± 8.2		0.7

**Table 4:** Univariate and multivariate predictors of VTI_QRS-3D_ in the overall healthy group.

Healthy group (n = 468)				
	Univariate		Multivariate*	

Variable	β-coefficient	p-value	β-coefficient	p-value
Age, years	−0.15 ± 0.02	**<0.0001**	−0.14 ± 0.02	**<0.0001**
Female	−6.56 ± 0.85	**<0.0001**	−6.41 ± 0.91	**<0.0001**
Race				
White	Ref	Ref	Ref	Ref
Black	1.79 ± 1.21	0.1	0.72 ± 1.14	0.5
Other	1.13 ± 1.15	0.3	−0.92 ± 1.09	0.4
Body surface area, kg/ m^2^	3.52 ± 1.57	**0.02**	−0.07 ± 1.61	1.0

* Multivariate model with predictors age (linear), female, race and body surface area (linear).

**Table 5A:** Distribution of VTI_QRS-3D_ among cardiomyopathy with reduced EF* females.

Variables (Females with cardiomyopathy)	n	VTI_QRS-3D,_ μVs Mean ± SD	IQR	Reference ranges 2.5th–97.5th percentile	*p*-value

Overall	108	48.5 ± 24.8	32.0–61.1	17.7–114.8	
**Age**					0.9
18–34	5	44.0 ± 11.9	32.6–49.4	31.4–58.4	
35–54	26	50.0 ± 27.8	32.3–71.8	18.4–110.6	
55–64	29	50.4 ± 25.8	36.7–59.8	22.4–120.8	
≥65	48	47.0 ± 24.0	29.6–61.7	18.7–99.3	
**Race**					0.3
White	62	45.4 ± 21.0	32.6–57.6	18.2–96.4	
Black	37	54.0 ± 29.6	32.2–71.6	19.6–117.5	
Other/unknown	9	47.0 ± 27.0	31.9–56.8	19.1–96.1	
**Cardiomyopathy type**					0.3
Ischemic	40	45.4 ± 22.2	29.3–60.9	18.5–100.0	
Non-ischemic	68	50.3 ± 26.3	32.6–61.5	18.3–114.8	
**Echocardiography and body composition**		**Mean ± SD**		**β-coefficient**	
Body surface area, m^2^	101	1.9 ± 0.3	−1.5 ± 7.5		0.8
LV internal dimension in diastole, cm	107	5.0 ± 0.9	12.0 ± 2.6		**<0.0001**
LV internal dimension in systole, cm	106	4.0 ± 1.0	12.5 ± 2.2		**<0.0001**
LV ejection fraction, %	108	35.4 ± 9.3	−0.7 ± 0.3		**0.01**
LV mass, g	107	196.2 ± 77.2	0.17 ± 0.03		**<0.0001**
LV mass indexed, g/m^2^	100	105.5 ± 38.3	0.40 ± 0.05		**<0.0001**
Interventricular septum, cm	108	1.0 ± 0.2	26.6 ± 9.5		**0.006**
LV posterior wall, cm	108	1.0 ± 0.2	42.6 ± 11.0		**0.0002**

* Left ventricular ejection fraction < 50 %.

**Table 5B:** Distribution of VTI_QRS-3D_ among cardiomyopathy with reduced EF* males.

Variables (Males with cardiomyopathy)	n	VTI_QRS-3D,_ μVs Mean ± SD	IQR	Reference ranges 2.5th–97.5th percentile	p-value

**Overall**	206	48.1 ± 19.4	33.1–58.8	21.7–98.3	
**Age, years**					0.2
18–34	9	61.9 ± 35.5	40.1–77.3	25.7–122.6	
35–54	54	49.1 ± 21.5	33.3–61.4	21.2–86.8	
55–64	54	46.9 ± 16.7	33.0–56.6	23.1–84.7	
≥65	89	46.9 ± 17.2	33.7–55.5	22.7–93.4	
**Race**					**0.007**
White	135	45.0 ± 16.5	32.7–54.4	21.8–86.7	
Black	42	53.7 ± 18.4	41.6–62.1	24.7–108.7	
Other/unknown	29	54.3 ± 28.7	33.2–63.9	22.3–129.7	
**Cardiomyopathy type**					**0.0003**
Ischemic	128	44.3 ± 17.3	32.4–50.5	21.7–86.9	
Non-ischemic	78	54.4 ± 21.2	40.1–63.4	25.4–110.6	
**Echocardiography and body composition**		**Mean ± SD**	**β-coefficient**		
Body surface area, m^2^	190	2.0 ± 0.3	−4.2 ± 5.1		0.4
LV internal dimension in diastole, cm	204	5.4 ± 0.8	4.5 ± 1.8		**0.01**
LV internal dimension in systole, cm	203	4.2 ± 0.9	6.3 ± 1.4		**<0.0001**
LV ejection fraction, %	206	36.2 ± 9.5	−0.3 ± 0.1		0.08
LV mass, g	204	231.9 ± 78.7	0.08 ± 0.02		**<0.0001**
LV mass indexed, g/m^2^	189	113.3 ± 34.7	0.20 ± 0.04		**<0.0001**
Interventricular septum, cm	204	1.1 ± 0.2	12.5 ± 5.7		**0.03**
LV posterior wall, cm	204	1.1 ± 0.2	20.6 ± 5.8		**0.0005**

* Left ventricular ejection fraction < 50 %.

**Table 6:** Univariate and multivariate predictors of VTI_QRS-3D_ in the cardiomyopathy* group.

Cardiomyopathy with reduced EF group (n = 314)				
	Univariate		Multivariate	

Variable	β-coefficient	p-value	β-coefficient	p-value
Age	−0.02 ± 0.09	0.8	−0.03 ± 0.09	0.8
Female	0.40 ± 2.55	0.9	0.23 ± 2.58	0.9
Race				
White	Ref	Ref	Ref	Ref
Black	8.69 ± 2.81	**0.002**	5.01 ± 2.73	0.07
Other	7.42 ± 3.73	**0.048**	5.68 ± 3.58	0.1
Body surface area	−2.98 ± 4.06	0.5	−2.47 ± 3.95	0.5
Type of cardiomyopathy				
Ischemic	Ref	Ref	Ref	Ref
Non-ischemic	7.92 ± 2.38	**0.001**	6.74 ± 2.46	**0.006**
LV ejection fraction (%)	−0.39 ± 0.13	**0.003**	−0.08 ± 0.13	0.5
LV mass indexed	0.27 ± 0.03	**<0.0001**	0.25 ± 0.03	**<0.0001**

* Left ventricular ejection fraction < 50 %.

**Table 7:** Reference ranges of VTI_QRS-3D_ in healthy group (N = 468).

VTI_QRS-3D_ in Healthy Population*						
Groups	n	Percentile				
		2.5th	25th	50th	75th	97.5th

**Females**						
18–34 years	92	23.2	32.6	37.6	44.3	55.0
35–54 years	107	23.9	30.5	34.6	41.2	56.4
≥ 55 years	100	19.60 .6	27.0	33.0	37.7	50.9
Overall	299	20.2	29.6	35.0	41.4	55.7
**Males**						
18–34 years	58	29.9	42.2	48.1	51.5	57.2
35–54 years	61	28.2	35.7	40.5	49.6	56.7
≥ 55 years	50	21.4	29.8	36.5	44.3	55.9
Overall	169	25.6	35.4	42.4	49.9	57.2
**Combined**						
18–34 years	150	24.1	34.5	41.7	48.3	57.1
35–54 years	168	23.9	32.0	37.5	44.7	57.3
≥ 55 years	150	18.9	28.1	34.3	40.7	55.3
Overall	468	20.9	31.5	37.3	45.2	56.4

* p < 0.0001 for two-sample t-test between females and males.
