# Supplementary material for: QRS 3D voltage-time integral in narrow QRS complex – Establishing the normal reference range
Source: J Electrocardiol. Author manuscript; Available in PMC 2025 Dec 3. (PMC12674847; doi:10.1016/j.jelectrocard.2025.154069)
Supplement: 1 [file NIHMS2112404-supplement-1.docx]

**Supplementary Table 1.** Comparison of SÂ QRS values by Pipberger et al.^*^ and 3D QRS area with difference method in healthy male population in our dataset.

| **Age group**  (years) | **Pipberger et al.^1^**  (N = 518, all males)  SÂ QRS (µVs) | **Healthy male population**  (n = 169)  3D QRS area using difference method (µVs) |
| --- | --- | --- |
| <20 | - | 25.9 ± 8.2 |
| 20-29 | 42.0 ± 13.0 | 29.9 ± 9.9 |
| 30-39 | 42.0 ± 14.5 | 29.2 ± 8.5 |
| 40-49 | 39.5 ± 13.0 | 25.6 ± 8.7 |
| 50-59 | 34.5 ± 11.4 | 24.4 ± 9.5 |
| 60-78 | 32.4 ± 13.4 | 24.1 ± 10.8 |
| >78 | - | 21.1 ± 9.5 |

* Pipberger HV, Goldman MJ, Littmann D, Murphy GP, Cosma J, Snyder JR. Correlations of the orthogonal electrocardiogram and vectorcardiogram with constitutional variables in 518 normal men. Circulation. 1967; 35:536-551. doi: 10.1161/01.cir.35.3.536

**Supplementary Table 2.** Comparison of SAI QRS values published by De la Garza Salazar et al.* and SAI QRS values from healthy population in our dataset.

| **Age group**  (years) | **De la Garza Salazar et al.^2^**  (N = 664) | **Healthy population**  (n = 468) | |
| --- | --- | --- | --- |
|  | SAI QRS (µVs) | | VTI_QRS-3D_ (μVs) |
| <60 | 64.7 ± 23.4 | 59.6 ± 14.4 | 39.0 ± 9.3 |
| ≥60 | 58.6 ± 20.2 | 53.3 ± 14.1 | 34.9 ± 8.9 |
|  | SAI QRS-T (µVs) | | VTI_QRST-3D_ (μVs) |
| <60 | 118.4 ± 41.1 | 120.7 ± 38.7 | 78.0 ± 24.2 |
| ≥60 | 106.2 ± 33.6 | 103.9 ± 28.2 | 67.3 ± 17.3 |

* De la Garza Salazar F, Egenriether B. Exploring vectorcardiography: An extensive vectocardiogram analysis across age, sex, BMI, and cardiac conditions. J Electrocardiol. 2024; 82:100-112. doi: 10.1016/j.jelectrocard.2023.12.004
